# Salbutamol Transport and Deposition in the Upper and Lower Airway with Different Devices in Cats: A Computational Fluid Dynamics Approach

**DOI:** 10.3390/ani11082431

**Published:** 2021-08-18

**Authors:** Rocio Fernández-Parra, Pascaline Pey, Carol Reinero, Mauro Malvè

**Affiliations:** 1 Department of Small Animal Medicine and Surgery, Faculty of Veterinary Medicine, Catholic University of Valencia “San Vicente Mártir”, Carrer de Quevedo, 2, 46001 Valencia, Spain; rocio.f.parra@ucv.es; 2 Pôle Anesthésie-Réanimation-Urgence-Soins Intensifs, Centre Hospitalier Universitaire Vétérinaire d’Alfort (CHUVA), École Nationale Vétérinaire d’Alfort, Université Paris-Est, 7 Avenue du Général de Gaulle, 94704 Maisons-Alfort, France; 3 Antech Imaging Services, 17620 Mt. Herrmann Street, Fountain Valley, CA 92708, USA; pascaline_pey@hotmail.fr; 4 Department of Veterinary Medicine and Surgery, University of Missouri, Columbia, MO 65211, USA; reineroc@missouri.edu; 5 Department of Engineering, Public University of Navarre (UPNA), Campus Arrosadía s/n, 31006 Pamplona, Spain; 6 Centro de Investigación Biomédica en Red en Bioingeniería, Biomateriales y Nanomedicina (CIBER-BBN), C/Poeta Mariano Esquillor s/n, 50018 Zaragoza, Spain

**Keywords:** asthma, chronic bronchitis, bronchodilator, feline model, pressurized metered dose inhaler, CFD

## Abstract

**Simple Summary:**

Administration of inhaled salbutamol via metered-dose inhalers can effectively treat bronchoconstriction. Different devices are used for the delivery of this drug in cats, either in the hospital or at home, for long-term treatment. Effective drug administration may depend on the drug delivery device as well as patient cooperation. By using non-invasive computational fluid dynamics techniques, the impact of these devices on the deposition and transport of salbutamol particles in the cat airways was simulated and assessed. The results confirm a variable drug distribution depending on the device used. The percentage of particles reaching the lung was reduced when using spacers and increased when applied directly into an endotracheal tube.

**Abstract:**

Pressurized metered-dose inhalers (pMDI) with or without spacers are commonly used for the treatment of feline inflammatory airway disease. During traditional airways treatments, a substantial amount of drugs are wasted upstream of their target. To study the efficiency of commonly used devices in the transport of inhaled salbutamol, different computational models based on two healthy adult client-owned cats were developed. Computed tomographic images from one cat were used to generate a three-dimensional geometry, and two masks (spherical and conical shapes) and two spacers (10 and 20 cm) completed the models. A second cat was used to generate a second model having an endotracheal tube (ETT) with and without the same spacers. Airflow, droplet spray transport, and deposition were simulated and studied using computational fluid dynamics techniques. Four regions were evaluated: device, upper airways, primary bronchi, and downstream lower airways/parenchyma (“lung”). Regardless of the model, most salbutamol is deposited in devices and/or upper airways. In general, particles reaching the lung varied between 5.8 and 25.8%. Compared with the first model, pMDI application through the ETT with or without a spacer had significantly higher percentages of particles reaching the lung (*p* = 0.006).

## 1. Introduction

Asthma and chronic bronchitis are common inflammatory airway disorders in cats. In people with asthma and chronic obstructive pulmonary disease (COPD; most commonly caused by chronic bronchitis and emphysema), the central and peripheral airways and lung parenchyma have characteristic pathologic lesions [1]. Similarly, in cats with spontaneous or experimental asthma and with chronic bronchitis, pathology centers on large airways (i.e., bronchi/bronchioles) and small airways (non-cartilaginous bronchioles < 2 mm in diameter) [2,3,4]. Parenchymal involvement was also documented in experimental feline asthma [2]. When employing therapy, the anatomic site of disease must be carefully considered. Bronchodilators such as inhaled salbutamol and corticosteroids are commonly administered to cats with inflammatory airway disease and, to be effective, must reach the affected regions of the lung.

Inhaled medications, including pressurized metered-dose inhalants (pMDI) allow high topical concentrations of affected airways while decreasing systemic side effects compared with oral or systemic routes of administration [5]. While the intent of inhaled medications in asthma and chronic bronchitis is to reach the large and small airways, substantial amounts of the drug are deposited in the oropharynx, nasal passages, nasopharynx, larynx, and trachea (herein collectively referred to as the upper airways) leading to drug waste and undesirable local high dosages [6]. Drug deposition not only depends on particle size but also on inhalation maneuver [7]. For this reason, knowledge of inhaled drug distribution within the respiratory tract using different drug delivery devices has relevance for pet cats. In humans, rising identification of problems associated with pMDI use, including uncoordinated inhalation and actuation technique, especially in pediatric patients, has led to modifications in the pMDI itself and the addition of auxiliary devices called spacers [8]. Different inhaler spacers have been specifically designed for cats (AeroKat™, Free-Space™, Nebulair™).

Rapidly acting bronchodilators are the most effective drugs during a life-threatening episode of bronchospasm in cats and, in this scenario, are administered either by inhalation or by injection. Salbutamol (or Albuterol), a selective short-acting beta-2 adrenergic agonist commercialized in a dose of 90–100 µg per inhalation, is the most commonly prescribed inhaled bronchodilator in cats. In comparison, terbutaline, another short-acting beta-2 adrenergic agonist, is generally recommended for use by injection and has shown efficacy in reversing bronchoscopy-induced airflow limitation [9]. In humans, a Cochrane review found no evidence to support requisite IV use of beta-2 agonists in patients with severe acute asthma and concluded that these drugs should be given by inhalation [10]. Direct comparison of inhaled and injectable bronchodilators in cats with severe bronchospasm has yet to be performed. Inhaled salbutamol is widely available and minimizes undesirable systemic cardiovascular changes as tachycardia, systemic hypertension, and others that occur with injectable bronchodilators [11].

The use of pMDI with a spacer has increased in cats and has become an important route for at-home treatment of feline bronchial disease [12]. However, in emergent clinical settings, when a spacer is not present, pMDI salbutamol is administered in different ways, including directly through a pre-oxygenation mask or directly through an endotracheal tube (ETT) in an intubated cat. The different administration techniques are expected to lead to different particle distribution and deposition of the drugs. 

Computational fluid dynamics (CFD) is a non-invasive numerical tool widely used in humans for studying a huge variety of problems, including aerosol transport and deposition [13]. Human studies using CFD have already compared particle deposition and transport depending on the techniques applied, devices used, and/or differences between healthy and asthmatics patients [14]. Idealized or patient-specific models have been used to predict particle lung depositions in humans [15,16]. These studies have offered relevant clinical information on inhaled drug aerosols’ therapeutic efficacy and/or the inhalation of polluted air. From computed tomography (CT) scans and magnetic resonance imaging (MRI) some authors created comprehensive numerical models from the oral cavity and several generations of bronchi or even the entire humans’ airways [15]. Little is known in animals, particularly in cats, and only a few studies were performed on rabbit [17,18], rat [19], and monkey [20] without clear clinical veterinary applications or just for improving human medicine and health.

Each type and formulation of the drug and delivery device must be optimized for the species and respiratory disorder of interest. The deposition and transport of salbutamol varies and depends on particle size, inspiratory flow velocity, and devices design used, among others. CFD technique was used to understand pMDIs performance, reduce drug waste, and improve the delivery of the aerosol particles to the lung considering different inhalers and devices [14]. In addition, the airflow varies throughout the respiratory system. In the upper airways, the flow is turbulent (Reynolds number > 2000), whereas as the airways narrow in the lower zones, the flow becomes laminar. This will also influence the behavior of the particles [7]. The CFD tool allows these flow regimes to be simulated.

In this study using healthy cats, our first aim was to assess the efficiency of pMDIs of salbutamol using different spacers, masks, and an ETT comparing particle deposition and transport in the respiratory tract of cats with numerical models. For reaching this goal, CT-based CFD models were created for determining particle deposition and/or transport in four regions: device, upper airways (nose to the trachea), primary bronchi, and downstream lower airways/parenchyma (herein referred to as “lung”). 

Our hypothesis was that by using CFD to model pMDI delivery of salbutamol in healthy cats, most particle deposition would take place in the drug delivery devices and the upper airways. We also hypothesized that particle transport would be enhanced in cats with an endotracheal tube compared with a spacer chamber or a pre-oxygenation mask. This study would help to better understand conditions for optimal pMDI drug delivery in healthy cats as a steppingstone to the investigation of cats with inflammatory airway disease such as asthma and chronic bronchitis. 

## 2. Materials and Methods

### 2.1. Animals

Two client-owned cats presented to the teaching veterinary hospital (Centre hospitalier universitaire vétérinaire d’Alfort—Chuva) between September 2019 and July 2020 with informed owner consent were enrolled in this study. One cat, without intubation, was under sedation for a head CT for otitis media and a second cat was intubated (ETT 4 ID) for a front limb CT which included the thorax. The weights of the cats enrolled were 4.25 and 4.5 kg, respectively. CT images were selected for geometrical reconstruction and further simulations.

### 2.2. Ethics

All procedures were conducted as part of normal veterinary clinical practice with owner’s consent form (Art. R242-48, Ordre National de Vétérinaire) and approved by the Clinical Research Ethical Committee of the École Nationale Vétérinaire d’Alfort (ENVA), France (Number: 2020-05-30).

### 2.3. Pressurised Metered Dose Inhaler (pMDI) and Devices Selection, CT Images, and Design

**Figure 1:**
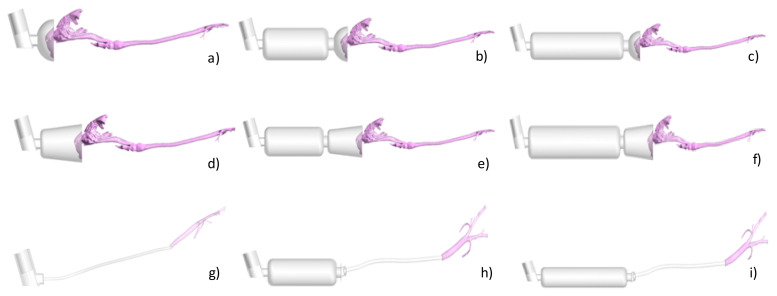
Pressurized metered-dose inhaler (pMDI) and device models for computational fluid dynamics simulations: (**a**) pMDI connected to a spherical preoxygenation mask, (**b**) pMDI connected to a 10 cm spacer and a spherical preoxygenation mask, (**c**) pMDI connected to a 20 cm spacer and a spherical preoxygenation mask, (**d**) pMDI connected to a conical preoxygenation mask, (**e**) pMDI connected to a 10 cm spacer and a conical preoxygenation mask, (**f**) pMDI connected to a 20 cm spacer and a conical preoxygenation mask, (**g**) endotracheal tube (ETT) 4 mm ID, trachea, and main bronchus, (**h**) pMDI connected to a 10 cm spacer and an ETT 4 mm ID, trachea, and main bronchus, (**i**) pMDI connected to a 20 cm spacer and an ETT 4 mm ID, trachea, and main bronchus. Computational CT-based models with Ansys: a–f, non-intubated cat model; g–i, intubated cat.

Nine different possible combinations of devices were geometrically reconstructed based on CT images or manufactured data (see Figure 1).

Using the CT study from the cats, a geometrical reconstruction was performed and then used for further analysis. The selected devices were two types of pre-oxygenation masks with spherical (Figure 1a) and conical (Figure 1d) shapes. Then, a spacer of 10 cm length and 4 cm diameter and a spacer of 20 cm length and same diameter were connected to the two previous masks (Figure 1b,c,e,f). For these CT images, no cats were required. Finally, a CT image of an intubated cat was selected with the aim of posteriorly creating a numerical model for simulating a direct administration of a pMDI through an endotracheal tube (Figure 1g) or through the two spacers (Figure 1h,i).

### 2.4. Sedation, Multidetector Computed Tomography (MDCT) Protocol and Image Analysis

Cats fasted for 12 h prior to anesthesia but had free access to water until premedication was administered. Sedation or general anesthesia was adapted to each cat. Cats were spontaneously breathing and placed in sternal recumbency with the head elevated and the neck fully extended. Non-contrast-enhanced MDCT examinations were carried out using a 64-detector-row CT system (Brilliance 64; Philips, Amsterdam, The Netherlands) before the programmed CT study. The CT images were obtained using a matrix of 768 × 768, tube voltage of 120 kV, tube current of 196 mA, and a display field of view of 35 cm and a pitch of 0.5. Images for the study were acquired first from the nostrils to the most caudal border of the lungs [21]. One-millimeter-thick images were reconstructed using a high-resolution algorithm. At the conclusion of the CT examination, the cat was supervised until completely recovered.

Images were reviewed using commercial medical imaging software, DICOM (Digital Imaging and Communications in Medicine, DICOM) viewer (Horos v.1.1.7., 64-bit, Horos^TM^, Brooklyn, NY, USA) using a lung window (window width (WW): 1600; window level (WL): −550). A board-certified radiologist performed the evaluation of the images for major abnormalities involving the airways.

### 2.5. Geometrical Reconstruction and Numerical Discretization

**Figure 2:**
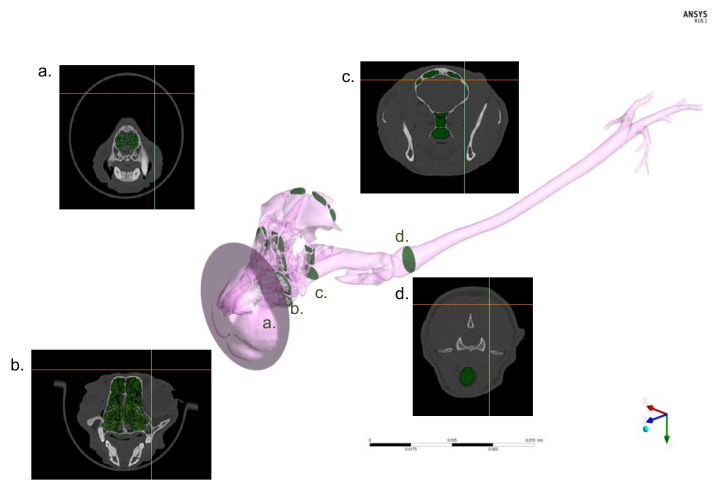
Computerized Tomography (CT)-based three-dimensional model generation. Non intubated cat is shown as an example: the four green markers correspond to transverse sections on CT from the rostral nasal cavity and muzzle (**a**), through the turbines meatus (**b**) and nasopharyngeal area (**c**) to the laryngeal entrance to the trachea (**d**). CT images are extracted from images and used with the other sections (not shown) for building the entire model (represented in purple).

The DICOM files derived from the CT studies were imported into the image-based geometry reconstruction software (MIMICS, Materialise Software, Leuven, Belgium; Figure 2).

Manual reconstruction of the upper airway (defined as the nasal cavity, nasopharynx, oropharynx, larynx, and trachea) and primary bronchi (also called principal or mainstem bronchi) geometry was conducted for the non-intubated cat.

**Figure 3:**
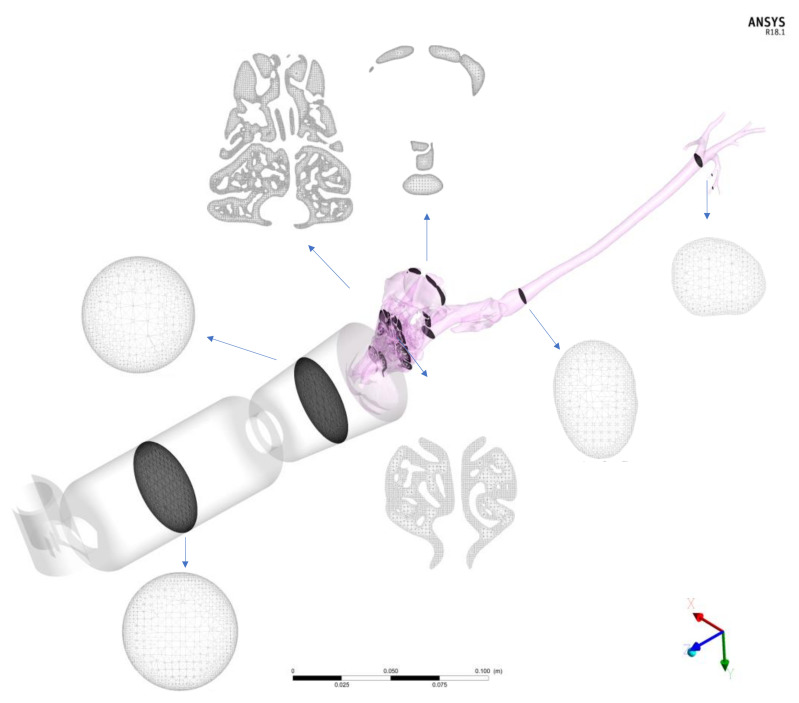
Representation of the numerical discretization in a non-intubated feline CT-based model of pressurized metered-dose inhaler (pMDI) drug delivery. The airways volumes are filled with three-dimensional elements (tetrahedrons). In this example, a pMDI is connected to a 10 cm spacer and a conical preoxygenation mask.

By means of the commercial software (Ansys IcemCFD, v.20, Ansys Inc., Canonsburg, PA, USA), device volumes and airways were filled with tetrahedral elements that composed the computational mesh in which the airflow governing equations, droplet formation, and droplet trajectories were solved. In Figure 3, a grid is shown for the spacer of 10 cm and the spherical mask using sections and corresponding images. 

Finally, two different masks of conical and spherical shape and two lengths of sacers as described previously were connected to the cat nose of the reconstructed model. The devices were created and attached to the patient-specific cat model in the commercial software package Rhinoceros (release 5, Robert McNeel and Associates, Seattle, WA, USA). The final model of the non-intubated cat is depicted in Figure 3.

The same procedure was used for the manual reconstruction of the intubated cat. In this case, only the trachea and the first generations of the respiratory tract were segmented, and the tracheal tube and the inhaler were separately generated and attached in Rhinoceros. The models are represented in Figure 1. In this case, the geometry is much less complex with respect to that of the other cat as it starts directly from the trachea bypassing all the nasal-to-laryngeal airways tract. Thus, the numerical grid contains far fewer elements resulting in less expensive computational costs. The volume was filled with three-dimensional elements (tetrahedrons; see Table 1), and the total number of elements varied depending on the presence or absence of spacers and the type of spacer. The number of tetrahedrons of the cat geometry within the non-intubated cats was approximately 18 million. The number of elements for the 10 cm and 20 cm length spacers ranged from 3 to 6 million, respectively. The number of elements for the spherical and conical masks ranged from 2.5 to 5 million, respectively. Prior to final computations, a mesh independence study was carried out in order to assess the dependence of the results on the grid size. Details of the study are provided in Appendix A.

### 2.6. CFD Analysis 

Once the devices and airways volumes were filled with tetrahedrons, the resultant numerical grids of the cats were imported into a simulation software package (Ansys CFX, v.20, Ansys Inc., Canonsburg, PA, USA). This software solves the Navier–Stokes equations that describe flow motion in different conditions within the geometrical grids using numerical algorithms. In particular, the Ansys CFX software adopts the finite volume method. The exact mathematical formulation and the solving algorithms used by Ansys CFX are provided in the software manual (Ansys, 2020).

**Figure 4:**
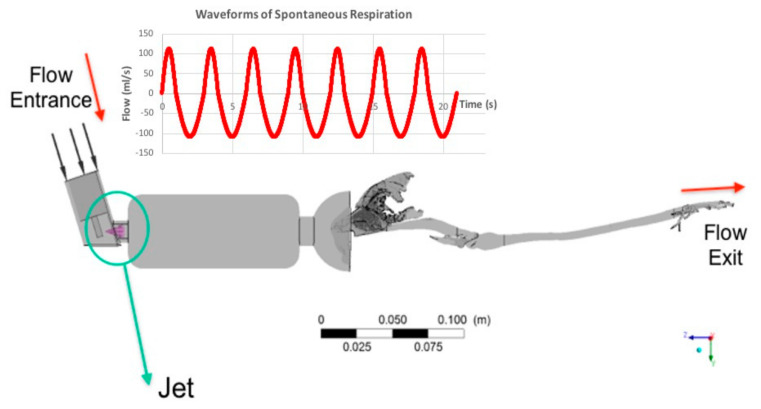
Pressurized metered-dose inhaler (pMDI) connected to a 10 cm spacer and a spherical preoxygenation mask model. Red arrows represent flow direction. Waveforms of spontaneous respiration are plotted in red in the upper part of the figure. The green circle and arrow highlight the location of the spray (in purple), nozzle orifice, and point of particle injection with a jet velocity of 150 m/s.

The peak inspiratory/expiratory flow of the non-intubated cat was 110 mL/s [22] and of the intubated cat, 30 mL/s. The flow of the intubated cat was obtained by means of a thermal anemometry (“hot wire”) flow sensor (Dräger Julian Anaesthesia Machine, Lübeck, Germany). The peak inspiratory flow was imposed at the top of the pMDI (see Figure 4). The flow was considered turbulent, and the k-ω model was used. An initial turbulence intensity value of 5% was adopted.

A respiratory cycle of 3 s (1 s inspiration, 2 s expiration) was selected and 7 respiratory cycles for a total of 21 s using a time step of 0.01 s were computed. Fluid properties, air density of 1.185 kg/m^3^, and viscosity of 1.83 10^–5^ Pa·s [21] were used. The pMDI modeling included a dose of 100 µg for puff, an initial droplet diameter of 10 µm, a velocity of 150 m/s, and a spray angle of 20° [23,24]. The pMDI puff was applied just before the first inhalation waveform starts in order to simulate clinical conditions.

The pMDI used in the model consists of a canister connected to a metering valve capable of producing required dosages of 25 to 100 µg. The user controls the release of the drug through the actuator-nozzle that generates a measured liquid. We used a nozzle orifice of 0.5 mm diameter and located at the center line of the canister in the center of the mask or the spacer (Figure 4).

The injected particles were then tracked through the geometric regions until they found one of the three specific conditions: (1) they collided and were trapped on the mask or spacer tube and/or on the airway walls, (2) they escaped from the domain through one of the outlet geometry faces, or (3) they continued in suspension in the flow. Suspension means that particles are still in a hold-up, and they could subsequently be exhaled or impact an airway wall in further breathings. The numerical models allow computing flow velocity and structures inside each cat’s airways and devices. The structure of the flow was depicted using 3D streamlines, while the flow intensity was represented using a heatmap.

All computations were performed on an Intel 9 workstation and parallelized on 8 processors. The computational costs of every single simulation are given in Table 1. Further details of the particle modeling and independent study are described in Appendix A and Appendix B [25,26,27].

### 2.7. Data Analysis

Airflow streamlines (peak inspiratory flow structures) and droplet deposition were represented using heat maps and described qualitatively for the various conditions in both models. Percentages of particles deposited to the devices, to the upper airways, and transported to the lung as previously defined for all conditions in each model, were reported. Further data analysis used IBM^®^ SPSS^®^ Statistics software version 23 (Chicago, IL, USA). A Shapiro–Wilk test was used to test for the normal distribution of percentages of particles deposited to the aforementioned three regions and in suspension for further comparisons. A *t*-test for independent values was used to compare the means and SD of the delivery percentages of particulates to devices, upper airways, to the lung, and in suspension, between the two models (i.e., the non-intubated cat and intubated cat). Values of *p* < 0.05 were considered statistically significant with a 95% of confidence interval (CI).

## 3. Results

**Figure 5:**
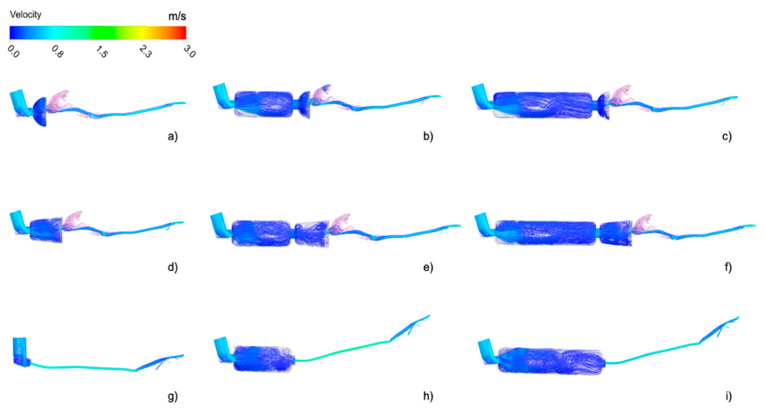
Peak inspiratory flow structure in the computed models depicted using streamlines and colored with the velocity intensity. (**a**) Spherical Mask, (**b**) Spherical Mask/Spacer 10 cm, (**c**) Spherical Mask/Spacer 20 cm, (**d**) Conical Mask, (**e**) Conical Mask/Spacer 10 cm, (**f**) Conical Mask/Spacer 20, (**g**) Endotracheal Tube (ETT), (**h**) ETT/Spacer 10 cm, (**i**) ETT/Spacer 20 cm. Red = high velocity; dark blue = low velocity.

Using CT scans from two cats, nine different combinations of the model devices were simulated. Airflow streamlines, which represent the flow direction depicted with the intensity of the velocity (red = high velocity, dark blue = low velocity) of all simulation models, are represented in Figure 5 at peak flow during inspiration.

**Figure 6:**
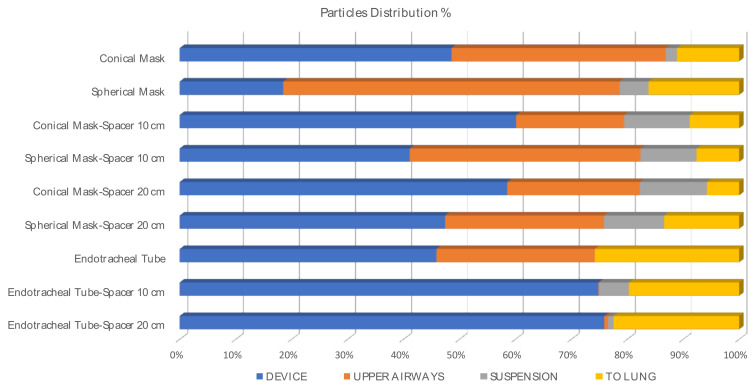
Percentage of particle distribution after 7 respiratory cycles (I:E ratio 1:2, inspiratory time 1 s) into the three regions (device, upper airways, to the lung), as well as remaining in suspension.

In the case of the spherical mask, the presence of the spacer seemed to have less influence on the flow. The recirculating airflow structure appeared similar independent of the presence of the spacer (Figure 5a–c). This may have been due to the geometry of the mask. The spherical mask was shorter than the conical mask hence producing a flow recirculation for its proximity to the cat nose in all the cases. In general, the presence of the spacers greatly influenced the flow in the masks, as visible in Figure 6, especially in the case of the conical mask (Figure 5d–f). The flow recirculation appearing in the conical mask in the presence of the spacer was largely reduced when the spacer was not attached. In fact, the air jet generated at the entrance of the conical mask was generated at the entrance of the two spacers when these were connected, and hence the flow velocity reduced, and its structure accordingly modifies.

The flow structure inside the spacer appeared similar within the models and independent of its length. After the skewed inlet of the inhaler ring, the airflow velocity in the spacer experimented with a sudden expansion at the spacer entrance, generating a local recirculation near the spacer walls. The airflow velocity increased again at the entrance of the mask and in the oral cavity and further accelerated in the trachea because of the constriction in the larynx. At this stage, the flow was completely turbulent.

The distribution of inhaled airflow was heterogeneous within the cat nasal cavity due to the complex geometry of the airways. The initial high-speed flow in these regions was slowed down in the dorsal meatus (Figure 5), progressively becoming a low-speed flow in the ventral regions (blue regions of Figure 5). Then, the flow was directed to the nasopharynx and laryngeal regions, where local acceleration occurs due to the physiological constriction represented by the larynx. Finally, the flow moved forward and divided to the lower airways through the bifurcations and generations from the carina.

The airflow in the intubated cat was more homogeneous in comparison to that of the non-intubated cat (Figure 5g–i). As the inhalation proceeded from the endotracheal tube, the airflow entered directly to the trachea and distributed to the airways with a lower velocity as the cat was anesthetized. No influence on the flow patterns was visible when the spacers were added to the tracheal tube. However, important variations were expected regarding the particle inhalation.

The total of particles in percentages (%) reaching the lung after seven respiratory cycles airways or remained in suspension after the simulation are represented in Figure 6 (I:E ratio 1:2, inspiratory time 1 s; see Table 2), attached to the device and/or upper.

Particle deposition percentage was computed as the ratio of deposited and injected particles times 100. These percentages are summarized for the inhaler, the spacers, and the masks in Table 3. They represent the amount of drug which is not able to reach the lungs.

Those particles deposited on the muzzle or in the upper airways (nasal turbines, oropharynx, larynx, and trachea) are represented in Table 4.

In the Supplementary Materials, the unsteady behavior of the droplets injected though the salbutamol pMDI are visualized during the breathing cycles (see Videos S1–S4).

The particle deposition in the devices was 2.6 times higher when a conical mask was used compared with a spherical mask alone. The drug deposition was twice when using a 20 cm spacer with a conical mask and 0.7 times higher when a 10 cm spacer was used compared to when the spherical mask was attached to them. However, the deposition was 1.6 times higher on the muzzle when using the spherical mask with respect to the conical one. Finally, 66.41% of the particles were deposited before reaching the upper airways in the conical mask and 48.96% in the spherical mask when they were used alone. When using these masks with a spacer of 10 cm that, as commented, is specific to cats, the particle deposition in the regions before the upper airways was found to be 22.65% and 26.85% (CM and SM, respectively). When a human pediatric spacer of 20 cm was used, these percentages were 41.35% (CM) and 30.15% (SM).

Mean and standard deviation (±SD) of the percentage of particles deposited into the devices, upper airways, and reaching the lungs (exit the computational model) when using a mask or an ETT with or without a spacer are represented in Table 5.

Mean and standard deviation (±SD) of the percentage of particles deposited into the devices, upper airways, and reaching the lung when a spacer was added or not are represented in Table 6.

**Figure 7:**
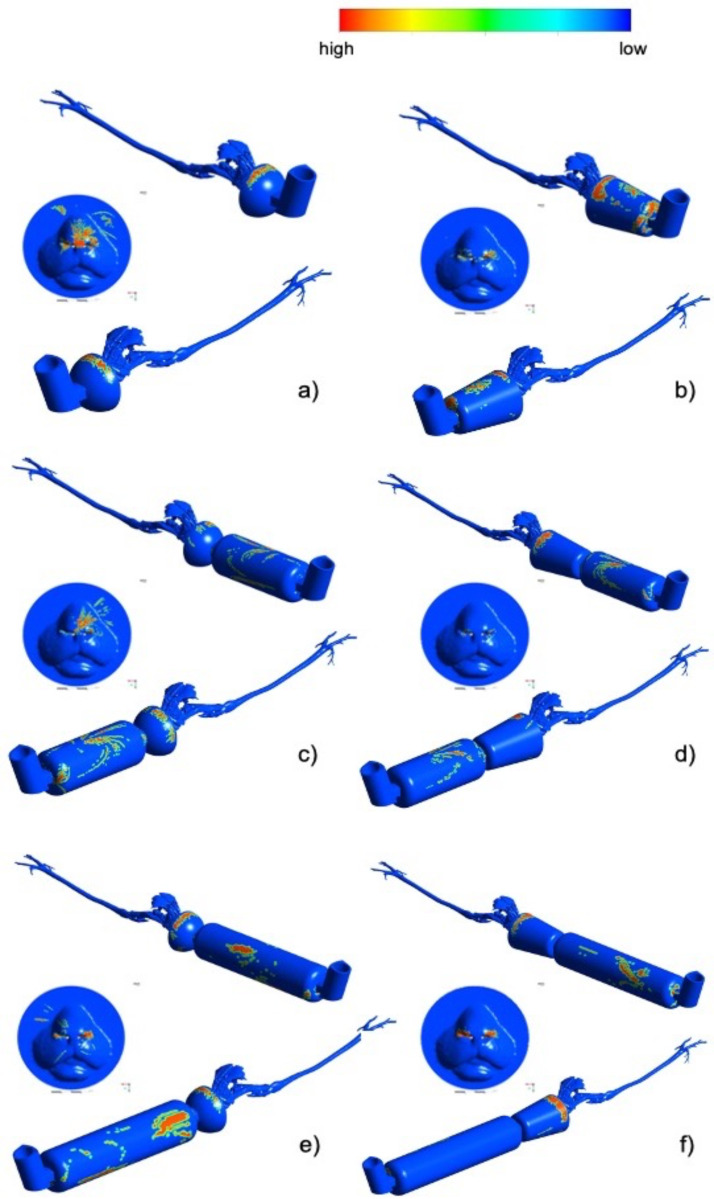
Salbutamol droplets deposition patterns depicted on the computational non-intubated cat models (right and left panels) after 7 respiratory cycles. (**a**) Spherical Mask, (**b**) Conical Mask, (**c**) Spherical Mask/Spacer 10 cm, (**d**) Conical Mask/Spacer 10 cm, (**e**) Spherical Mask/Spacer 20 cm, (**f**) Conical Mask/Spacer 20. In each subfigure, two longitudinal views are represented. Deposition on the cat muzzle is also represented. Red = high; dark blue = low or zero droplets deposition.

In Figure 7, the salbutamol droplets deposition patterns are represented on the geometrical model surfaces. The pattern provided a qualitative picture of the regions affected by high or low salbutamol concentration. Visible droplets tend to deposit in non-uniform ways. There was a tendency of particles to deposit especially in the upper mask region (see Figure 7a,b). When the spacer was introduced into the model, the deposition on the mask was slightly reduced. A considerable amount of salbutamol was concentrated on the spacer surface (see Figure 7c–f). Salbutamol deposition on the muzzle was enhanced when using the spherical mask. In any case, salbutamol loss due to deposition was not significantly different between particles attached to the devices or upper airways (*p*= 0.428 and *p* = 0.089, respectively).

## 4. Discussion

The present study aimed to assess the transport, distribution, and deposition of salbutamol 10 µm particles in the upper and lower airways of cats by means of the CFD technique. The pMDI application through the ETT, with or without a spacer, had significantly higher percentages of particles reaching the lung compared with the non-intubated model. In humans, similar studies were used to improve aerosol delivery techniques with similar aims, and the use of the CFD technique is widely known [15,23,28,29]. However, compared with humans, the anatomy of cats’ upper airways is more complex and thus challenging for this technique. Studies assessing deposition of aerosol clinical therapies in small animals are limited and based on the use of Scintigraphy in dogs [30]. The use of pertechnetate (^99m^Tc) delivered via a spacer and face mask apparatus in conscious cats has been published [31], but this technique employs the use of radioisotope and gamma-rays, which is more invasive and may be toxic for the patient. Less invasively, Leemans et al., 2009, compared the duration of action and the efficacy of ß2-agonists in cats with induced bronchoconstriction using barometric whole-body plethysmography (BWBP) [32]. The advantage of this numerical tool is that it is a non-invasive technique. 

We simulated the most commonly prescribed bronchodilator at home and used in clinics, salbutamol, with a concentration of 100 µg per puff (Ventolin, GlaxoSmithKline plc (GSK), Brentford, UK). It is the same product with exactly the same dosage as the one used in humans (average weight of 70 kg). It is unknown what plasma level is required to achieve effective bronchodilatation in cats, and toxicity trials with aerosolized formulations in cats are missing. Leemans et al., 2009, are the only group that studied the effects and justified a dose for salbutamol pMDI. Their study recommended a single dose of 100 µg with a peak effect of around 15 min post administration and inducing an antispasmodic effect in the airways for up to 4 h. Two- or four-fold increments of drug dosages slightly improve bronchodilatory effects. Similar results were reported in humans [33,34]. Consequently, in this study, a single dose of 100 µg was simulated, with a fixed peak inspiratory flow for the non-intubated and intubated model. 

Treatment recommendations varied between the time of application and/or the number of breaths after activation of the pMDI [34]. Some companies recommend allowing the cat to breathe through the mask for 10 to 15 s or to take 5 to 10 breaths after activation of the pMDI (Trudell Medical International, London, ON, Canada). In this study, seven respiratory cycles for a total of 21 s (physiological I:E ratio of 1:2) were imposed since we wanted to allow enough time to make sure that all the particles were distributed into the model. Even so, in most cases, there was still a percentage of particles that remained in suspension, except for the direct pMDI endotracheal tube (ETT) administration. In this case, after less than one complete respiratory cycle (after 1.1 s), all particles were already distributed. It is also worth mentioning that for the conical mask, only 2% of the particles remained in suspension. Hence with ETT and conical mask alone, particles were transported faster.

The peak inspiratory/expiratory flow was lower in the simulated intubated cat (30 mL/s) compared with the non-intubated cat (110 mL/s). Significant greater percentages reaching the lung were found in this study when applying pMDI directly in the ETT (19.7–25.8%) compared with the other techniques (5.75–16.2%; *p* = 0.006), as preconized in our hypothesis. In humans, the serum concentrations of an inhaled drug are greater in intubated patients as there is no oropharyngeal deposition, no enteral absorption, and best lung deposition [35]. One-third of the total aerosol output per pMDI puff is expected in smaller ETT size (4 mm ID as was used in the current study) compared with >5 mm ID ETT [36]. Side effects after ß2-agonist administration directly in the ETT during general anesthesia were described in horses. Specifically, sinus tachycardia, premature ventricular complexes or/and hypotension [37], and sweating [38] were observed after the administration of aerosolized salbutamol. As there are no reports in cats, the exact plasma concentration at which bronchodilation is expected versus at which side effects will appear is unknown, and thus this practice should be employed with caution. Side effects in cats may include tremors, central nervous system (CNS) excitement, vomiting, mydriasis, and/or dizziness [39].

Flow velocities and patterns affect particle behavior and hence their deposition and transport [40]. It was suggested that the use of a spacer might reduce particle velocity from the pMDI and may allow a better distribution [8]. Other studies found that an add-on spacer with a pMDI may result in a considerable reduction in the number of respirable particles available to the patient [41]. The clinical significance of this effect is not well established in humans [7,42]. Although a spacer was specifically designed for cats (AeroKat^®^, Trudell Medical International, London, ON, Canada), some veterinarians and/or clients still choose the option of pediatric/baby spacers. These spacers are usually longer and may or may not have a one-way valve (valved holding chambers, VHC). The dose delivered may vary considerably between spacers, and this has to be considered when changing from one spacer to another [43]. For this reason, we have included both types of spacers in the current study. Our results suggest that no benefit is produced by the use of one or the other type of spacer (Figure 5), independently of the type of mask. The percentage of particles reaching the lung was similar in the presence and in the absence of a spacer (*p* = 0.757). This may be due to the flow structures inside the masks, which revealed high recirculation. The Global Initiative for Asthma (GINA) recommends the use of pMDI and a dedicated spacer/VHC with facemask for children aged 4 years and younger and a pMDI plus a dedicated spacer/VHC with mouthpiece for children aged between 4 and 6 years. In comparison, for adult humans, no mask is added to the spacer to maximize the spacer’s function of reducing particle velocity yet improving particle transport [23]. No guidelines or consensus around this topic exists in veterinary medicine. In this study, more particles were deposited in the masks when the spacer was not added, probably as the spray nozzle was nearer (see Table 3 and Table 4). In any case, more particles remained in suspension when using a spacer (*p* = 0.037). That is, even after 21 s, there were still particles that had not reached any structure or the lung. It is possible that when using a spacer, more time is needed for the salbutamol to fully reach the lungs. However, the total number of particles transported to the lungs was similar between all groups. 

Particles reaching the lung using pMDI in humans vary considerably between studies. From 11 to 14%, drug deposition was reported in ambulatory patients using radiolabeled aerosols [34]. Other studies suggest that less than 10% of the inhaled bronchodilator finally reaches the target area when using pMDI alone [44,45], and from 10% to 38% with the use of the pMDI plus a spacer [46]. In a computational study using a salbutamol pMDI with a spacer attached to a human upper airway model and a flow velocity of 30 L/minute, 52.9% of particles traveled to the lung [23]. In the present study, the percentage of particles reaching the lung was higher than 10% in most of the cases and varied between 5.75 and 25.8% depending on the device used. Most of the studies considered particles sizes from 1 to 10 µm [29,40]. In humans, it was reported that the deposition also depends on the particle size [29]. In particular, while nanoparticles deposition tends to increase when the particle size decreases (<0.1 µm), deposition of microparticles tends to increase when the size increases (>1 µm). 

Reduced lung deposition percentages will be expected in clinical situations, where cats do not tolerate excessive handling, particularly when respiratory distress is present. This is in comparison to the current study’s models in which the devices fit perfectly with the cat’s anatomy, and all the particles are utilized. The deposition fractions summarized in Table 3 suggest that spherical masks may enhance particle transport into the lungs compared to conical masks. However, in the presence of the spherical mask, the deposition in the rostral area, on the muzzle, and in the nasal passage is higher than in the presence of the conical mask (see Table 4). Additional studies, including a greater number of cat models, are needed for a better comparison between masks.

Other factors that may modify particle lung deposition when using pMDI aerosol in ventilated humans are the ventilator mode, settings, and circuit [47], and synchronization of pMDI application with breaths, among other factors [48]. In addition, the therapeutic response may be influenced by the patient’s airway anatomy and disease severity [49]. Further studies are needed to determine all these factors in cats. 

The results that can be extracted from the computational analyses have to be considered carefully. The computational models, in general, are affected by unavailable limitations. The main limitation of this study was that an ideal and perfect fitting mask with or without a spacer was simulated, where 100% of the particles are sent to the cat airways. This may not reflect reality. Another limitation concerned the limited number of animals. Further studies including additional animals are necessary. However, the aim of the study was to compare different devices and not different cat anatomies. For this reason, only two cats were used to develop the models. Finally, only particles of 10 µm size were simulated. Smaller particle diameters should also be investigated as their behavior strongly depends on size, as was demonstrated in humans [29].

## 5. Conclusions

Using CT scans to develop feline models and CFD to investigate salbutamol transport and deposition, this study determined that most of the particles deposited in the devices and/or upper airways before reaching the lung. Direct administration of pMDI through the ETT has the largest and fastest lung deposition compared with the rest of the devices. The use of a pediatric spacer versus a specifically designed spacer for cats did not impact droplets transport. Further studies using a larger number of cats, patient-specific airflows, and different particle sizes are warranted to investigate which delivery devices may have better and safer performance.

## Figures and Tables

**Table 1:** Computational cost and numerical discretization of each model.

Device	Computational Cost (Hours)	Grid Size (Millions)
Conical Mask	167.6	23
Spherical Mask	152.5	20.5
Conical Mask/Spacer 10 cm	200.4	26
Spherical Mask/Spacer 10 cm	169.4	23.5
Conical Mask/Spacer 20 cm	271.1	29
Spherical Mask/Spacer 20 cm	197.3	26.5
ETT	18.3	4
ETT/Spacer 10 cm	48	7
ETT/Spacer 20 cm	68.5	10

ETT: Endotracheal Tube.

**Table 2:** Percentage of particles reaching the lung after 7 respiratory cycles (I:E ratio 1:2, inspiratory time 1 s).

Device	Total
Conical Mask	11.11
Spherical Mask	16.2
Conical Mask/Spacer 10 cm	8.85
Spherical Mask/Spacer 10 cm	5.75
Conical Mask/Spacer 20 cm	7.616
Spherical Mask/Spacer 20 cm	13.4
ETT	25.8
ETT/Spacer 10 cm	19.7
ETT/Spacer 20 cm	22.45

All values are described in percentages (%). ETT: Endotracheal Tube.

**Table 3:** Percentage of particles deposited to the different parts of the devices.

Device	Inhaler	Spacer	Mask	Total
Conical Mask	0.58	-	48.01	48.59
Spherical Mask	0.015	-	18.51	18.51
Conical Mask/Spacer 10 cm	0.8	43.3	16.05	60.15
Spherical Mask/Spacer 10 cm	0.075	29.25	11.8	41.125
Conical Mask/Spacer 20 cm	0.15	33.4	25	58.55
Spherical Mask/Spacer 20 cm	0.15	35.2	12.1	47.45
ETT	45.9	-	-	45.90
ETT/Spacer 10 cm	0.65	74.2	-	74.85
ETT/Spacer 20 cm	0.85	74.2	-	75.90

All values are described in percentages (%). ETT: Endotracheal Tube.

**Table 4:** Percentage of particles deposited to the different areas of the upper airways.

Device	Muzzle	Nasal Turbines	Oropharynx	Larynx	Trachea	Total
Conical Mask	18.4	19.89	0	0.01	0.01	38.31
Spherical Mask	30.45	29.25	0.25	0.15	0.2	60.30
Conical Mask/Spacer 10 cm	6.6	12.60	0.05	0.05	0.05	19.35
Spherical Mask/Spacer 10 cm	15.05	24.75	0.95	0.5	0.05	41.30
Conical Mask/Spacer 20 cm	16.35	7.20	0.01	0.05	0.14	23.75
Spherical Mask/Spacer 20 cm	18.05	10.3	0	0.05	0	28.40
ETT	-	-	-	-	28.3	28.3
ETT/Spacer 10 cm	-	-	-	-	0.1	0.1
ETT/Spacer 20 cm	-	-	-	-	0.65	0.65

All values are described in percentages (%). ETT: Endotracheal Tube.

**Table 5:** Percentage of particle distribution and deposition comparing when using a mask or an ETT with or without a spacer.

Particles	Group	*n*	Mean ± SD	*p*-Value	95% CI
Deposited in the devices	Mask	6	45.73 ± 15.14	0.428	−31.158 12.450
	ETT	3	55.08 ± 17.13		
Deposited in the upper airways	Mask	6	35.24 ± 14.87	0.089	0.188 45.872
	ETT	3	12.21 ± 19.95		
Reaching the lung	Mask	6	10.44 ± −3.85	0.006 ^a^	−17.052 −5.696
	ETT	3	21.81 ± 4.66		
In suspension	Mask	6	8.56 ± 4.08	0.061	0.733 11.087
	ETT	3	2.69 ± 2.67		

Lung refers to lower conducting airways distal to the primary bronchi to the parenchyma. ETT: Endotracheal Tube; CI: confidence interval; SD: Standard Deviation; Super index letter (a), means significant differences between groups (*p* < 0.05).

**Table 6:** Percentage of particle distribution and deposition when using or not a spacer.

Particles	Group	*n*	Mean ± SD	*p*-Value	95% CI
Deposited in the devices	Spacer	6	54.44 ± 12.59	0.131	−35.982 2.441
	No	3	37.67 ± 16.65		
Deposited in the upper airways	Spacer	6	19.03 ± 15.99	0.051	4.297 46.880
	No	3	44.62 ± 13.67		
Deposited in the lung	Spacer	6	13.67 ± −8.25	0.757	−8.43 11.742
	No	3	15.33 ± 3.92		
In suspension	Spacer	6	8.76 ± 3.81	0.037 ^a^	−11.239 −1.521
	No	3	2.38 ± 2.59		

ETT: Endotracheal Tube; CI: confidence interval; SD: Standard Deviation; Superscript letter (a) means significant differences between groups (*p* < 0.05); No = without spacer.

**Figure A1:**
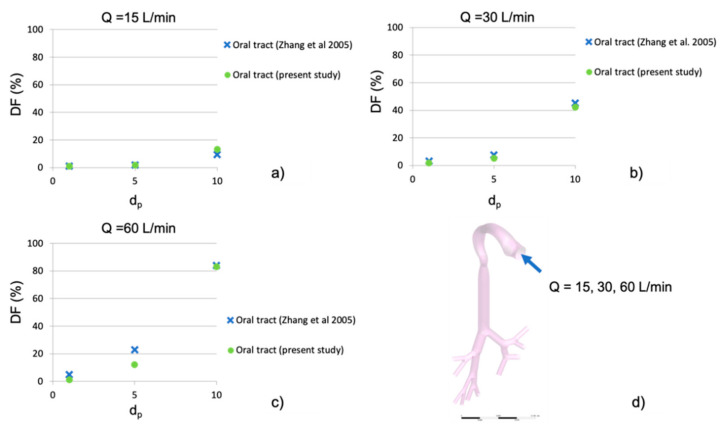
Computational human model used for validation: deposition fractions (DF; in %) are computed and displayed as a function of the particle diameter d_p_ (in µm). Inspiratory flows Q of 15, 30, and 60 L/min are used for simulating light, normal, and heavy activities. Comparison between literature and the human idealized computational model of the aerosol deposition fractions as a function of the particle diameter at (**a**) Q = 15 L/min, (**b**) Q = 30 L/min and (**c**) Q = 60 L/min. In (**d**), the geometrical model used for the simulations is shown with the different breathing rates Q (blue arrow).

**Figure A2:**
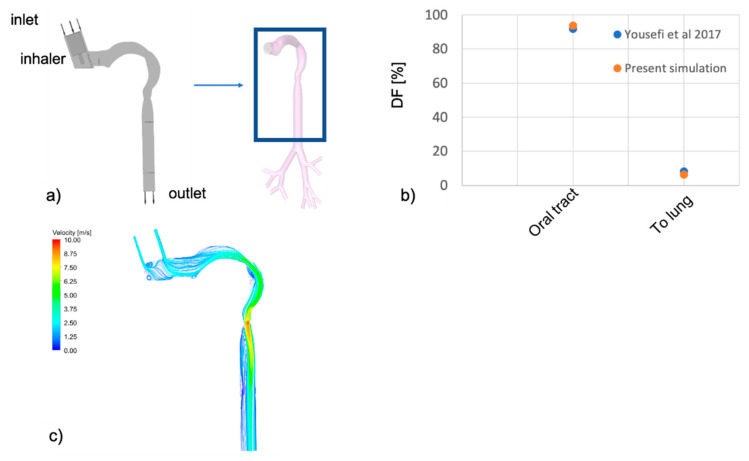
Computational human model with human upper airway tract and pMDI used for validation (**a**) and regional deposition fractions (DF; in %) computed and displayed in comparison with the literature data (Yousefi et al., 2017) for inspiratory flows Q of 30 L/min (**b**). In (**c**), the velocity distribution inside the oral airways is represented by means of velocity streamlines.

## Data Availability

The data generated during the current study are not publicly available because they are part of a national research project. However, some data could be available from the corresponding author on reasonable request.

## Supplementary Materials

The following are available online at https://www.mdpi.com/article/10.3390/ani11082431/s1, Video S1: Unsteady simulation of the particle deposition and transport within the non-intubated cat model with spherical mask. Video S2: Unsteady simulation of the particle deposition and transport within the non-intubated cat model with conical mask. Video S3: Unsteady simulation of the particle deposition and transport within the non-intubated cat model with spherical mask and spacer of 10 cm length. Video S4: Unsteady simulation of the particle deposition and transport within the non-intubated cat model with conical mask and spacer of 10 cm length.

## Appendix A

### Appendix A.1. Governing Equations

The fluid dynamics model presented in this study is based on the work presented by Kleinstreuer and co-workers [23] and Inthavong and co-workers [24] in humans, and it is well known and widely used for numerical simulations of particle transport and deposition in the human airways. For this reason, here we provide only a summary of the main features of the model, and additionally, validation with the literature data is presented.

The fluid dynamics of the cat breathing subjected to the turbulence and specific boundary conditions were solved simultaneously with the droplet formation and particle trajectories. The latter was solved using Newton’s second law governed by drag and gravity as the major forces for dispersed droplets in low-density gas flow [23]. For solving the viscous and incompressible airflow in the cat airways, the well-known Reynolds averaged Navier–Stokes equations were used.

$$\nabla \cdot \bar{v}=0$$

$$\frac{d\bar{v}}{dt}+\nabla \cdot \left(\bar{v}\times \bar{v}\right)=-\frac{1}{\rho }\;\nabla p^{\prime }+\nabla \cdot \left(\upsilon_{eff}\left(\nabla \bar{v}+{\left(\nabla \bar{v}\right)}^{T}\right)\right)$$

where $\bar{v}$ is the averaged fluid velocity vector, *ρ* is the gas density, and *p*′ is modified pressure, *v_eff_* is the effective kinematic viscosity (*v_eff_* = *v* + *v*_t_, where *v* is gas kinematic viscosity, and *v*_t_ is turbulence kinematic viscosity). Between the kinematic and the turbulent kinematic viscosities exists the relation *v*_t_ = *k*/ω.

The turbulence was modeled using the Wilcox *k*-ω model [26]. This model predicts the turbulence using two partial differential equations for the variable *k* and ω, where *k* represents the turbulence kinetic energy and ω is the rate of dissipation of the turbulence kinetic energy. Both equations are provided below:

$$\frac{\partial k}{\partial t}+\nabla \cdot \left(vk\right)=\nabla \cdot \left[\left(v+\frac{v_{t}}{\sigma_{k}}\;\right)\nabla k\right]+P_{k}-\beta 'k\omega$$

$$\frac{\partial \omega }{\partial t}+\nabla \cdot \left(v\omega \right)=\nabla \cdot \left[\left(v+\frac{v_{t}}{\sigma_{\omega }}\;\right)\nabla \omega \right]+\alpha \frac{\omega }{k}P_{k}-\beta \omega^{2}$$

where the term *P_k_* that represents the turbulent production can be obtained as:

$$P_{k}=v_{t\;}\nabla v\cdot \left(\nabla v+{\left(\nabla v\right)}^{T}\right)$$

and *α* = 5/9, *β* = 0.075, *β′* = 0.09 and *σ_k_* = *σ_ω_* = 2 are turbulent constants [26].

### Appendix A.2. Model of the Salbutamol Spray

The droplet spray was simulated with gas–liquid interactive Enhanced Taylor Analogy Breakup (ETAB) model [23]. With this model, a particle exposed to gas flow is subjected to large drag force by the surrounding gas if a velocity difference between particle and gas exists. The drag force produces droplet formations accordingly to the Weber number (ratio of the inertia force to surface tension):

$$We=\rho \frac{v^{2}r}{\sigma }$$

where *ρ* is the air density, *v* is the relative velocity between air and particle, *r* is the particle radius, and *σ* is the coefficient of liquid surface tension. This non-dimensional ratio connects droplet deformation and the liquid surface tension. Particle trajectories are determined by CFX using Newton’s second law in the presence of drag force and gravity. This equation can be expressed as:

$$m_{p}\frac{\;dv_{p}}{dt}=\frac{1}{8}\pi \rho d_{p}{}^{2}c_{Dp\;}\left|v-v_{p}\right|\left(v-v_{p}\right)+g$$

where ***v****_p_* is the particle velocity, *m_p_* is the particle mass, rho is the gas density, ***g*** is the gravitational force, *c_Dp_* is the drag coefficient, and *d_p_* is the particle diameter. The drag coefficient *c_Dp_* can be obtained with the relation:

$$c_{Dp}=\frac{c_{D}}{C_{slip}}$$

where *C_slip_* is the Cunningham correction factor and *c_D_* can be obtained as follows:

$$c_{D\;}=\max\left(\frac{24}{Re_{p}}\left(1+0.15Re_{p}{}^{0.687}\right),\;0.44\right)$$

where *Re_p_* is the particle Reynolds number given by:

$$Re_{p}=\;\frac{\left|v-\;v_{p}\;\right|\;d_{p}}{\upsilon }$$

where *v* is the kinematic viscosity of the gas.

Particles of 10 μm were injected through the nozzle orifice with the aforementioned velocity and the described spray properties and modeling.

### Appendix A.3. Mesh and Particle Independence Study

Due to the complex geometry of the cat’s upper airways, unstructured tetrahedral computational grids were created using the commercial software Ansys ICEM CFD, Version 20.0 (ANSYS Inc., Canonsburg, PA, USA). Tetrahedral elements filled the 3D airways volumes with a variable number and dimensions of elements. For this reason, to ensure independent results from the selected mesh element size, a mesh independence study was carried out. For this study, we considered the biggest geometry volume that is the cat model with the conical mask and the spacer of 20 cm. The model volume was discretized with an increasing number of elements, and by using each generated mesh, a CFD solution was obtained. We tested several different grids from 10 to 50 million elements. After the analysis of the velocity profiles at different geometrical cross-sections, it was clearly seen that from a grid size of around 30 million elements, the results were grid-independent.

The final model contains about 15 million tetrahedrons in the cat airways, about 6 million tetrahedrons in the spacer, about 5 million tetrahedrons in the mask, and about 4 million for the remaining model objects. As the mesh independence study estimates the necessary regional size, the other geometries used the same discretization technique. Of course, depending on the presence or absence of the spacer, and its length, and on the mask and its type, the global grid size changed. In other words, all geometries were previously subdivided into regions in which the value of the tetrahedron can be specified. For obtaining all the grids in all the analyzed cases, we used the same values obtained in the independent grid for each region of the considered models. As the mesh independence study was carried out on the biggest geometry (the spacer of 20 cm and the conical mask are the biggest devices), the grid sizes of the rest of the models were smaller. In particular, it is worth reporting that the grid size of the spherical mask was about 2.5 million tetrahedrons, and the spacer of 10 cm was composed of about 3 million tetrahedrons. The element sizes per each model that were used for the presented simulations are reported in Table 1.

A particle number-independence study was also conducted in order to verify that 2000 injected particles were statistically sufficient for describing the salbutamol transport and deposition. Since every single particle of 2000 injected is representative of a certain number of particles, it should be proved that the selected number correctly describes the physical phenomenon in the study. The geometry selected with this aim is the same used for the mesh independence study (spacer of 20 cm and conical mask). The CFD simulation was repeated using the same grid size but releasing a different number of particles. Particles exiting the geometries and depositing on the different regions of the airways were systematically collected, increasing the injected number to 200,000. No relevant changes in percentage were observed.

## Appendix B

### Validation of the Numerical Methodology with Literature Data

To validate the result obtained with the presented simulations, the computational methodology used for creating the in silico models needs to be validated. With this aim, and because no information is available in cats, spherical microparticle deposition was studied in a human upper airways geometry and compared with experimental and computational literature data (see below). For this, we used the typical idealized “path lung model” [26] widely known and used in the human computational aerosol technique. The comparison, at different breathing rates, for different particle diameters, and for the mentioned model are shown in Figure A1. In particular, particle diameters of 1, 5, and 10 µm were injected into the model through the mouth at light, normal, and heavy conditions (i.e., 15, 30, and 60 L/min). Steady inspiratory flows were computed, and particles deposited or exited from the model were collected. The results show that the model set-up is correct as it is capable of reproducing the results presented by Zhang et al. [29].

Additionally, the human upper airways in the presence of the pMDI and of the aerosol spray were simulated. The deposition fractions within the upper airways and the fraction of particles reaching the lung were compared with the results obtained by Yousefi et al. [24]. The latter presents a model of the airflow, droplet spray transport, and deposition in a pMDI attached to a human upper airway model, considering de Ventolin as a propellant. The model described in the present work includes a human oral cavity described in the previous Section B1 and a trachea truncated at its bottom. A pMDI model, as described by Kleinstreuer et al. [23], was attached to the mouth or to the model entrance (see Figure A2). The following boundary conditions were given to the model: an inspiratory flow rate of Q = 30 L/min and a turbulence intensity value of 5% for the turbulence kinetic energy was applied to the canister ring (see Figure A2); a pressure condition was applied to the outlet (bottom section of the trachea); a perfect absorption to the model walls for the particles. At the inhaler nozzle, a droplet initial diameter of 10 µm was assigned with a spray velocity of 110 m/s and a spray angle of 10° [24]. Finally, the used Ventolin density was 1230 kg/m^3^ with an actuation dose of 100 µg [24]. Again, deposited particles were collected at the oral tract (mouth + soft palate + pharynx + larynx + trachea), and particles traveling to the lung were computed. The comparison with the data presented by Yousefi et al. demonstrates that again the methodology used in the present work is correct, and the model is capable of reproducing the literature results.
